# The relationship between Internet addiction and childhood trauma in adolescents: The mediating role of social support

**DOI:** 10.3389/fpsyg.2022.996086

**Published:** 2022-10-05

**Authors:** Xuanlian Sheng, Meng Yang, Menglin Ge, Ling Zhang, Cui Huang, Shu Cui, Qiuyu Yuan, Mengting Ye, Ruochen Zhou, Panpan Cao, Ran Peng, Kai Zhang, Xiaoqin Zhou

**Affiliations:** ^1^School of Mental Health and Psychological Sciences, Anhui Medical University, Hefei, China; ^2^Department of Psychiatry, Chaohu Hospital of Anhui Medical University, Hefei, China; ^3^Anhui Psychiatric Center, Anhui Medical University, Hefei, China

**Keywords:** Internet addiction, childhood trauma, social support, mediating effect, adolescents

## Abstract

**Background:**

Adolescents are at high risk of Internet addiction (IA). Previous studies have shown that the occurrence of IA may be associated with childhood trauma and social support to varying degrees. This study aimed to investigate the pathogenesis of IA in adolescents. Further, to explore the potential mediating role of social support in childhood trauma and IA. This study provides theoretical support for future interventions targeting IA in adolescents.

**Methods:**

This study used a descriptive cross-sectional design. The Childhood Trauma Questionnaire (CTQ), Social Support Rating Scale (SSRS), and Young’s Internet Addiction Test (IAT) were used to conduct an anonymous questionnaire survey among randomly selected adolescents aged less than 18 years old in two cities in southern Anhui Province. Descriptive statistics and correlation analysis were used to test the correlation between IA and other variables. A binary logistic regression model was used to explore the influencing factors of IA. Multiple regression models were examined with process macro and bootstrapping to confirm significant mediating effects.

**Results:**

A total of 844 adolescents, equally divided between males and females, participated in this study, and the prevalence of IA in the region was 23.0%. Through the mediation test, the direct effect of childhood trauma on IA was 0.20 (95% CI [0.12, 0.27], *p* < 0.001), and the mediating effect of social support on childhood trauma and IA was 0.09 (95% CI [0.06, 0.14]).

**Conclusion:**

The study showed that childhood trauma significantly affected the incidence of IA in adolescents. Social support had a significant mediating effect on childhood trauma and IA and attenuated its negative effects.

## Introduction

The Internet is gradually affecting people’s psychology and behavior as a new way of life. Adolescents spend more time than adults establishing and maintaining social interactions on the Internet (Tsitsika et al., 2009). The 49th Statistical Report on China’s Internet Development shows that as of December 2021, the number of Internet users under the age of 19 years reached 182 million, accounting for 17.6% of the overall number of 1.032 billion Internet users (China Internet Network Information Center [CINIC], 2021). Internet addiction (IA), an impulse-control disorder, usually refers to a persistent and recurrent maladaptive behavior, causing distress and significant functional impairments (Young, 1998). Due to a lack of consensus on the conceptualization and measurement instruments for IA, the prevalence of IA among adolescents ranges from 0.4% to 44.7% worldwide (Müller et al., 2015; Kawabe et al., 2016; Vigna-Taglianti et al., 2017; Chung et al., 2019; Chi et al., 2020b; Chia et al., 2020; Xu et al., 2020; Bickham, 2021). The increasing prevalence of IA is related to adolescents’ low emotional stability and poor self-regulation ability (Sasmaz et al., 2014; Dong et al., 2021). IA is accompanied by physical and psychiatric symptoms, such as depression, anxiety, and loneliness, while forcibly stopping or reducing Internet use may cause a withdrawal reaction (Tao et al., 2008). Previous literature has found that many factors are related to IA, including age, sex, left-behind status, family economic status, etc. (Shek et al., 2008; Wu C. S. T. et al., 2016; Wang et al., 2019).

One of the models that have been influential in the IA field in recent years is the Interaction of Person-Affect-Cognition-Execution (I-PACE) model, which posits that biopsychological factors, such as early childhood experiences, contribute to IA (Brand et al., 2016, 2019). Studies have shown that adverse childhood experiences, such as childhood trauma, can negatively affect the developing brain, reducing the ability to inhibit memory and control, further leading to IA (Brand et al., 2016; Jhone et al., 2021). Childhood trauma usually refers to psychological and physical harm suffered during childhood, including emotional abuse, physical abuse, sexual abuse, emotional neglect, and physical neglect (Kessler et al., 2010; Zhang et al., 2020). Childhood trauma is the major predictor of DSM-IV lifetime disorders (Kessler et al., 2010). Researchers support the idea that IA is linked to different types of childhood trauma including childhood physical abuse, emotional neglect, and emotional abuse (Zhang et al., 2012; Kwak et al., 2018; Emirtekin et al., 2019; Kircaburun et al., 2019). The rate of IA among people with childhood trauma is 1.5 times higher than that of the general population (Yang et al., 2017).

Lack of social support as part of social cognition in the I-PACE model leads to excessive Internet use, which may eventually develop into IA (Brand et al., 2016). Social support was found to have a possible relationship with childhood trauma and IA (Brand et al., 2016; Negriff et al., 2019). Abused children who grow up in families with dysfunctional parents may not feel supported by their families, and reduced support may lead to higher rates of IA (Lo et al., 2021). Although childhood trauma is associated with poorer social support, good social support is one mechanism that may buffer the negative effects of childhood trauma, although this has not been shown in all studies (Appleyard et al., 2010; Negriff et al., 2015). Good social support plays a mediating role in the adverse effects of early life stress, brings positive psychological factors, and may reduce the occurrence of IA (Negriff et al., 2019). When social connections are low in the real world, it is easier for adolescents to establish intimate relationships in the Internet world, acquire a sense of belonging, and indulge in games to release their emotions, which increases the risk of IA among adolescents (Jia et al., 2018).

Childhood trauma and social support play important roles in the development of IA, but the role of social support between the two is unclear. This study surveyed a random sample of selected primary and secondary schools to understand adolescents’ general IA status. Following this, the specific relationship among IA, childhood trauma, and social support was explored. Finally, whether social support plays a mediating role in the relationship between childhood trauma and IA was explored. This study provides theoretical support for the prevention of IA in adolescents.

## Materials and methods

### Participants

This study was a cross-sectional investigation. From October 2020 to October 2021, researchers randomly selected 7 schools in Xuancheng City and Chaohu City in Anhui Province, and then randomly selected 4 classes (accounting for about 15% of all students in the school) in each school for a paper questionnaire survey. A total of 924 questionnaires with complete general information were collected. All adolescents were under 18 years of age and had no major physical or mental illness. Informed consent was obtained from all participants and their guardians before the investigation. Using the unified instruction language, the researchers gave unified instructions to the participants. The questionnaire was completed anonymously, and the participants were required to complete the questionnaire carefully and independently. The Ethics Committee approved this study at Chaohu Hospital, Anhui Medical University (2019-kyxm-012).

### Measurements

#### General sociodemographic data

Data on the participants’ age, sex grade, whether they were an only child, family economic status, accommodation type, parental marital status, parental education level, and left-behind status were collected.

#### Young’s Internet addiction test

We used Young’s Internet Addiction Test (IAT) to determine pathological Internet use (Young, 1998; Karaer and Akdemir, 2019; Panova et al., 2021). It contains 20 items in total. The scale adopts a five-point scoring method, with items scored from not at all (one) to always (five). The total score ranges from 20 to 100. Participants who score less than 50 points are considered not to be addicted to the Internet, and those who score more than 50 points are considered to be addicted to the Internet (Young, 1998; Cao et al., 2011; Tan et al., 2016; Xu et al., 2020). We used the Chinese version for evaluation, which had good reliability and validity (Cronbach’s α coefficient = 0.93) (Lin et al., 2009; Cao et al., 2011; Tan et al., 2016; Chi et al., 2020a).

#### Childhood trauma questionnaire short form

The Childhood Trauma Questionnaire Short Form (CTQ-SF) is used to assess traumatic life experiences during childhood (Bernstein et al., 1994; Georgieva et al., 2021). There are 28 items in the CTQ-SF, divided into the following five subscales, with five items per subscale: emotional abuse, physical abuse, sexual abuse, emotional neglect, and physical neglect. Each entry is rated on a scale of 5, with responses of never, occasionally, sometimes, often, and always. According to the manual of the retrospective self-evaluation questionnaire, scores ≥ 13 on the emotional abuse subscale, ≥ 10 on the physical abuse subscale, ≥ 8 on the sexual abuse subscale, ≥ 15 on the emotional neglect subscale, and ≥ 10 on the physical neglect subscale, are considered to indicate moderate to severe childhood trauma, as long as one subscale score meets the above conditions (Bernstein et al., 2003). The Chinese version of the CTQ-SF was translated and revised and proved to have good reliability, validity, and internal consistency (Zhao et al., 2005). The Cronbach’s α coefficient value was 0.743.

#### Social support rating scale

The Chinese Social Support Rating Scale (SSRS) is based on foreign scales, according to the actual situation in China, and includes the subjective support, objective support, and support utilization domains (Xiao, 1994). In our study, the terms colleagues and leaders in the questionnaire were changed to classmates and teachers to make the scale suitable for use with adolescents (Luo et al., 2017; Yu et al., 2018). In total, the scale consists of 10 items, with scores ranging from 1 to 4 for each item. A higher total score indicates a higher level of social support for adolescents. The SSRS showed moderate internal consistency with a Cronbach’s α coefficient of 0.812.

### Statistical analysis

SPSS 23.0 software was used for the statistical data analysis in this study. According to the total IA score, IA was converted into a dichotomous variable and divided into the IA group (total score ≥ 50) and the non-IA group (total score < 50). All data were tested for normality using the Shapiro–Wilk test. General sociodemographic data were analyzed using the independent sample *t*-test. The childhood trauma scale subscales were all converted into dichotomous variables according to the scale calculation method, and the chi-square test was applied to compare the differences between groups for each subscale. The difference in social support between the two IA groups was also tested using an independent samples *t*-test. Pearson correlation was used to analyze the correlation among the three variables. Binary logistic regression was used to analyze the influence of general sociodemographic data, childhood trauma, and total social support scores on IA. Using process 3.3 to analyze the mediating effect of social support between childhood trauma and IA, bias-corrected 95% confidence intervals (CIs) were calculated using a bootstrapping resampling of 5,000, and indirect effects were considered significant if the bootstrapped 95% CI did not include zero. Differences were considered statistically significant at *p* < 0.05 (two-tailed).

## Results

### Comparison of general sociodemographic data between Internet addicted users and non-Internet addicted users

Based on the above criteria, we collected 924 questionnaires, of which 9 were missing for the IAT, 37 were missing for the CTQ-SF, and 33 were missing for the SSRS. Ultimately, a total of 824 valid questionnaires were collected, with questionnaires from 428 males and 416 females. Their average age was 12.96 (1.49) years. The results of the comparison of general sociodemographic data between groups are shown in Table 1. Most of the participants were junior high school students (41.7%), non-only children (59.6%), and commuting students (77.3%); their parental marital status was married (83.2%); their family economic status was general (73.0%); and most of their parents had a junior high school education or below (60.8%, 64.9%).

**TABLE 1:** Comparison of general sociodemographic data between the Internet addiction group and the non-Internet addiction group.

Variables	Total sample (*N* = 844) (%)	IA (*n* = 194) (%)	Non-IA (*n* = 650) (%)	t/χ^2^	*p*
Age (x ± s)	12.96 ± 1.49	13.48 ± 1.49	12.81 ± 1.46	5.58	**<0.001**
Sex				3.02	0.08
Male	428 (50.7%)	109 (56.2%)	319 (49.1%)		
Female	416 (49.3%)	85 (43.8%)	331 (50.9%)		
Grade				34.61	**<0.001**
Primary school	334 (39.6%)	46 (23.7%)	288 (44.3%)		
Junior high school	394 (46.7%)	103 (53.1%)	291 (44.8%)		
High school	116 (13.7%)	45 (23.2%)	71 (10.9%)		
Only child or not				0.19	0.66
Yes	341 (40.4%)	81 (41.8%)	260 (40.0%)		
No	503 (59.6%)	113 (58.2%)	390 (60.0%)		
Parental marital status				14.40	**0.001**
Married	702 (83.2%)	147 (75.8%)	555 (85.4%)		
Divorce	130 (15.4%)	46 (23.7%)	84 (12.9%)		
Death of a parent	12 (1.4%)	1 (0.5%)	11 (1.7%)		
Family economic status				9.37	**0.009**
Better	180 (21.3%)	29 (14.9%)	151 (23.2%)		
General	616 (73.0%)	148 (76.3%)	468 (72.0%)		
Poor	48 (5.7%)	17 (8.8%)	31 (4.8%)		
Accommodation type				43.68	**<0.001**
Boarding student	192 (22.7%)	78 (40.2%)	114 (17.5%)		
Commuting student	652 (77.3%)	116 (59.8%)	536 (82.5%)		
Father’s education level				3.49	0.17
Junior high and below	513 (60.8%)	129 (66.5%)	384 (59.1%)		
High school	263 (31.2%)	51 (26.3%)	212 (32.6%)		
University and above	68 (8.1%)	14 (7.2%)	54 (8.3%)		
Mother’s education level				3.76	0.15
Junior high and below	548 (64.9%)	137(70.6%)	411 (63.2%)		
High school	235 (27.8%)	44 (22.7%)	191 (29.4%)		
University and above	61 (7.2%)	13 (6.7%)	48 (7.4%)		
Left-behind status				26.11	**<0.001**
Yes	421(49.9%)	128 (66.0%)	293 (45.1%)		
No	423 (50.1%)	66 (34.0%)	357 (54.9%)		

Bold values in the table indicate *p* < 0.05 (two-tailed).

There were statistically significant differences between groups in age, grade, parental marital status, family economic status, accommodation type, and left-behind status (*p* < 0.05). Compared to the non-IA group, a greater percentage of males had IA; more than half were junior high school students. Adolescents whose parental marital status was divorced had a significantly higher percentage of IA. A poorer family economic status also increased the percentage of IA. The proportion of boarding student in the IA group was also significantly more than twice as large as that in the non-IA group. The proportion of adolescents with left-behind experiences in the IA group was also more than half of that in the non-IA group. In addition, although there was no significant difference between groups regarding parental education level and whether they were only children; it was observed that the proportion of IA was higher in families with non-only adolescents and parents with lower education levels.

### Comparison of childhood trauma and social support between the Internet addiction group and the non-Internet addiction group

As shown in Table 2, all subitems and total scores were statistically significant (*p* < 0.05). More than half of the adolescents (*n* = 578, 68.5%) had experienced at least one type of childhood trauma. The percentage of adolescents who felt emotionally neglected reached 55.0%, while less than half of those with the other four types of traumatic experiences identified themselves as traumatized. IA was more common among adolescents with at least one traumatic childhood experience (*n* = 164, 84.5%) than among adolescents with non-traumatic childhoods. Except for emotional neglect, the proportion of children in the IA group with childhood trauma included in the other categories of childhood trauma was approximately two times higher than that of the adolescents in the non-IA group. The non-IA group had higher mean scores on all subscales of social support.

**TABLE 2:** Comparison of childhood trauma and social support scale between the Internet addiction group and the non-Internet addiction group.

Variables	Total (*N* = 844) (%)	Non-IA (*n* = 650) (%)	IA (*n* = 194) (%)	χ^2^/Z	*P*
Emotional abuse				26.46	<0.001
No	668 (79.1%)	540 (83.1%)	128 (66.0%)		
Yes	176 (20.9%)	110 (16.9%)	66 (34.0%)		
Physical abuse				14.39	<0.001
No	754 (89.3%)	595 (91.5%)	159 (82.0%)		
Yes	90 (10.7%)	55 (8.5%)	35 (18.0%)		
Sexual abuse				15.98	<0.001
No	782 (92.7%)	615 (94.6%)	167 (86.1%)		
Yes	62 (7.3%)	35 (5.4%)	27 (13.9%)		
Emotional neglect				17.37	<0.001
No	380 (45.0%)	318 (48.9%)	62 (32.0%)		
Yes	464 (55.0%)	332 (51.1%)	132 (68.0%)		
Physical neglect				30.38	<0.001
No	455 (53.9%)	384 (59.1%)	71 (36.6%)		
Yes	389 (46.1%)	266 (40.9%)	123 (63.4%)		
Childhood trauma				30.08	<0.001
No	266 (31.5%)	236 (36.3%)	30 (15.5%)		
Yes	578 (68.5%)	414 (63.7%)	164 (84.5%)		
Total social support	37.37 (6.62)	38.20 (6.35)	34.60 (6.76)	6.83	<0.001
Subjective support	20.55 (3.62)	21.01 (3.43)	19.02 (3.83)	6.91	<0.001
Objective support	9.14 (2.50)	9.23 (2.43)	8.82 (2.70)	2.02	0.044
Support utilization	7.68 (2.28)	7.94 (2.29)	6.78 (2.00)	6.40	<0.001

### Correlation analysis of childhood trauma, social support, and Internet addiction

Table 3 shows a correlation between two of the three variables. There was a negative correlation between IA and social support (*r* = −0.23, *p* < 0.01). Childhood trauma was positively associated with IA (*r* = 0.25, *p* < 0.01). Social support and childhood trauma were also negatively associated (*r* = −0.45, *p* < 0.01), but subjective support and objective support were not significantly associated with sexual abuse.

**TABLE 3:** Correlation coefficient between social support, childhood trauma, and Internet addiction.

Variables	I	II	III	IV	V	VI	VII	VIII	IX	X	XI
Internet addiction (I)	1										
Objective support (II)	−0.07^b^	1									
Subjective support (III)	−0.23^a^	0.40^a^	1								
Support utilization (IV)	−0.22^a^	0.35^a^	0.49^a^	1							
Total social support (V)	−0.23^a^	0.71^a^	0.86^a^	0.75^a^	1						
Physical neglect (VI)	0.22^a^	−0.22^a^	−0.32^a^	−0.29^a^	−0.36^a^	1					
Emotional neglect (VII)	0.12^a^	−0.25^a^	−0.40^a^	−0.31^a^	−0.42^a^	0.49^a^	1				
Physical abuse (VIII)	0.13^a^	−0.09^a^	−0.19^a^	−0.18^a^	−0.19^a^	0.30^a^	0.25^a^	1			
Emotional abuse (IX)	0.24^a^	−0.15^a^	−0.32^a^	−0.28^a^	−0.33^a^	0.37^a^	0.36^a^	0.57^a^	1		
Sexual abuse (X)	0.16^a^	–0.04	–0.06	−0.07^b^	−0.07^b^	0.25^a^	0.18^a^	0.55^a^	0.42^a^	1	
Childhood trauma (XI)	0.25^a^	−0.24^a^	−0.42^a^	−0.37^a^	−0.45^a^	0.69^a^	0.79^a^	0.65^a^	0.73^a^	0.54^a^	1

^*a*^Correlation is significant at the 0.01 level (two-tailed).

^*b*^Correlation is significant at the 0.05 level (two-tailed).

### Risk factors for Internet addiction

Combined with the above data analysis, the independent variables with statistical significance were included in the regression analysis to explore the influencing factors of IA and the total score of the two scales. Age, grade, parental marital status, family economic status, and accommodation type were added as covariables to establish a regression equation model (Table 4). After collinearity diagnosis, the variance inflation factor values of each variable in this regression model were less than 10, indicating that there was no collinearity. The result showed that accommodation type (OR = 0.47, 95% CI = 0.28–0.81), left-behind status (OR = 0.61, 95% CI = 0.42–0.88), and total social support score (OR = 0.93, 95% CI = 0.90–0.96) were hindering factors of IA; childhood trauma (OR = 1.92, 95% CI = 1.21–3.04) was a contributing factor to IA. Adolescents with high social support were less likely to have IA than those with lower social support. Adolescents with childhood trauma were more likely to have IA than adolescents without childhood trauma. In other words, adolescents without childhood trauma or with higher levels of social support were less likely to develop IA.

**TABLE 4:** Risk factors for Internet addiction.

Variables	B	SE	Wals	*p*	OR	VIF	95%CI	
							Lower	Upper
Age	0.20	0.12	2.91	0.09	1.22	3.96	0.97	1.53
Grade	−0.06	0.30	0.05	0.83	0.94	5.35	0.52	1.69
Parental marital status	0.25	0.20	1.57	0.21	1.28	1.05	0.87	1.89
Family economic status	0.04	0.19	0.05	0.82	1.04	1.11	0.72	1.52
Left-behind status	−0.50	0.19	7.02	**0.01**	0.61	1.13	0.42	0.88
Accommodation type	−0.75	0.27	7.62	**0.01**	0.47	1.93	0.28	0.81
Total Score for Social Support	−0.07	0.01	24.91	**0.00**	0.93	1.18	0.90	0.96
Childhood trauma	0.65	0.23	7.74	**0.01**	1.92	1.18	1.21	3.04
Constant	−0.58	1.51	0.15	0.70	0.56			

SE, standard error; OR, odds ratio; VIF, variance inflation factor; CI, confidence interval. Bold values in the table indicate *p* < 0.05 (two-tailed).

### The mediating effect of social support on childhood trauma and Internet addiction

**FIGURE 1:**
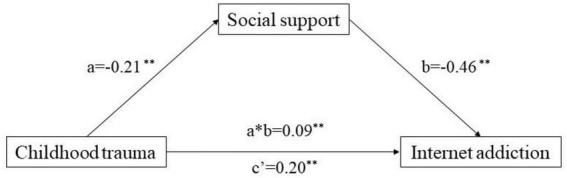
The mediating effect of social support on childhood trauma and Internet addiction (a*b, indirect effect; c, total effect; c’, direct effect; **p < 0.001).

There were significant correlations among social support, childhood trauma, and IA (Tables 5, 6 and Figure 1). To further explore the relationship among the three variables, Y represents IA as the dependent variable, X represents the total childhood trauma score as the independent variable, and M represents the total social support score as the intermediate variable to test the mediating effect. The possible effect was tested by multiple regression analysis after controlling for demographic variables (age, grade, parental marital status, family economic status, left-behind status, accommodation type, etc.).

**TABLE 5:** Coefficients of path analysis of variables.

Model pathways		Coefficients	*R* ^2^	*F*	*p*	95%CI	
						Lower	Upper
X→M		−0.21(*a*)	0.22	34.32	<0.001	–0.24	–0.18
X→Y		0.29(*c*)	0.19	27.96	<0.001	0.22	0.36
X→M→Y	X→Y	0.20(*c*′)	0.22	29.68	<0.001	0.12	0.27
	M→Y	−0.46(*b*)			<0.001	–0.62	–0.31

X, childhood trauma; Y, Internet addiction; M, social support. Adjusted for age, grade, parental marital status, family economic status, left-behind status, and accommodation type.

**TABLE 6:** Direct and indirect effects and 95% confidence intervals for the modal.

	Effect size	SE	LLCI	ULCI	Relative effect value
Total effect (c)	0.29	0.03	0.22	0.36	
Direct effect (c’)	0.20	0.04	0.12	0.27	68.97%
Indirect effect (a*b)	0.09	0.02	0.06	0.14	31.03%

Adjusted for age, grade, parental marital status, family economic status, left-behind, and accommodation type.

Statistical analysis showed that childhood trauma (*p* < 0.001) and social support (*p* < 0.001) had an influence on IA, and childhood trauma (*p* < 0.001) also had an effect on social support. The direct effect of childhood trauma on IA was 0.20 (SE = 0.04, *p* < 0.001, 95% CI = 0.12–0.27), and the mediating effect of social support in the relationship between childhood trauma and IA was significant, with a mediating effect value of 0.09 and an effect share of 31.03%.

## Discussion

This study mainly explored the relationship among IA, childhood trauma, and social support in adolescents. The prevalence of IA among adolescents in this area was 23.0% and 84.5% of them had at least one childhood trauma experience. Social support can reduce the risk of IA. At the same time, the mediating model showed that social support mediated the effect of childhood trauma on IA to some extent, and the mediating effect of social support accounted for 31.03%.

There were differences in the general sociodemographic data of adolescents in this region. The average age and grade of the IA group were older and higher, similar to previous studies (Shek et al., 2008). Men are more likely to be addicted to the Internet than women, but the proportion of men who are addicted to the Internet is higher than that of women. Convenient access to the Internet may weaken the sex differences (Seyrek et al., 2017). Incomplete family structures, such as families with divorced parents and lower family income, predict a higher risk of IA (Wu C. S. T. et al., 2016; Chi et al., 2020b). They have limited time to build relationships with their adolescents, who use the Internet to express their psychological insecurities and as a substitute for real life (Ni et al., 2009; Wu C. S. T. et al., 2016). Parents also fail to prevent IA in time (Wu C. S. T. et al., 2016). Left-behind adolescents are more likely to develop IA (Wang et al., 2019; Cai et al., 2021). The main reasons may be as follows: on the one hand, the Internet provides adolescents with an opportunity to escape negative events (Xin et al., 2018); on the other hand, the Internet also serves as a substitute for parental affection. Living on campus also increases the risk of IA due to a lack of regulation (Tang et al., 2020).

This study found that childhood trauma was positively correlated with IA and had a positive predictive effect on IA (Dalbudak et al., 2014; Yang et al., 2017; Hou et al., 2021; Hsieh et al., 2021). Similar to the results of previous studies, the severity of physical neglect and emotional neglect has a more significant impact on IA than the other three variables (Dalbudak et al., 2014; Schimmenti and Bifulco, 2015). While much previous research has focused on the devastating impact of physical and sexual abuse on adolescents, the emotional aspects of trauma cannot be ignored and may even have a greater impact. With the progress and development of society, corporal punishment of adolescents may be less than before. Nevertheless, parents being busy with work and a lack of companionship may cause adolescents to experience a sense of physical and psychological neglect. The findings may have something to do with how parents today educate their adolescents. Traumatized adolescents who are raised inappropriately, and live in unsafe home, environments are at high risk for mood disorders and may exhibit addictive behaviors, such as excessive Internet use as self-therapy for negative emotional problems (Schimmenti et al., 2014; Schimmenti and Bifulco, 2015; Hsieh et al., 2021).

Some studies of depressive symptoms in adolescents confirmed that more traumatic childhood experiences were associated with higher levels of depressive symptoms and, in turn, less perceived social support (Ji et al., 2017; Negriff et al., 2019). Childhood trauma was negatively correlated with social support. Adolescents who experience trauma and had more supportive social interactions were less likely to have poor mental health and tended to be healthier with more sources of support, making them less likely to become addicted to the Internet (Forster et al., 2020; Schneider et al., 2020). Social support was also negatively correlated with IA. All aspects of social support differed between the two groups, with support from friends and family particularly important for adolescents’ emotional and social development. When adolescents do not have sufficient social support, they may feel emotionally rejected, experience increased loneliness, and have decreased self-esteem. Therefore, these adolescents meet their social requirements through the Internet and develop alternative social relationships to increase their self-esteem and seek self-affirmation (Wu X. S. et al., 2016; Karaer and Akdemir, 2019; Ban, 2021); the Internet has become a cathartic outlet. Adolescents who are addicted to the Internet are at higher risk of IA due to their weak personal and family relationships, resulting in social isolation, loneliness, depression, family disharmony, academic failure, and even bullying by their peers (Wu X. S. et al., 2016).

The limitations of this study are as follows: First, this study was a cross-sectional study, making it difficult to determine causality, and further validation by cohort studies is needed. Second, this study only collected data from a few random schools in two cities of Anhui, which may not be widely representative, and data from other regions need to be further collected. Finally, the scales are all self-rated, and there may be some recall bias for childhood traumatic experiences.

## Conclusion

In conclusion, childhood trauma affects IA directly or indirectly through social support. Social support mediates the role of childhood trauma on IA. After suffering from childhood trauma, adolescents are prone to emotional disorders. With reduced support, these adolescents have no place to talk to others and seek help. They turn to the Internet to seek comfort from strangers or indulge in games to experience emotional release. It also suggests that the first step in preventing Internet addiction is to avoid childhood trauma as much as possible, which creates a vicious cycle leading to addictive behavior. Thus, the prevention of IA in adolescents can be managed hierarchically according to the situation (Kwak et al., 2018); family therapy programs can be provided to better prevent and reduce the risk of IA by coordinating relationships between family members (Kwak et al., 2018), increase the sources of social support for adolescents, and improve the utilization of support to alleviate the negative emotions caused by trauma and reduce the risk of IA.

## Data availability statement

The raw data supporting the conclusions of this article will be made available by the authors, without undue reservation.

## Ethics statement

The Ethics Committee approved this study at Chaohu Hospital, Anhui Medical University (2019-kyxm-012). Written informed consent to participate in this study was provided by the participants’ legal guardian/next of kin. Written informed consent was obtained from the individual(s), and minor(s)’ legal guardian/next of kin, for the publication of any potentially identifiable images or data included in this article.

## Author contributions

XS, MY, and MG designed and wrote the manuscript. XS, MY, MG, LZ, CH, SC, QY, MTY, RZ, PC, and RP collected the data. XS and KZ analyzed the data. XZ verified the data. All authors read and agreed to the final manuscript.
